# The incidence and aetiology of hospitalised community-acquired pneumonia among Vietnamese adults: a prospective surveillance in Central Vietnam

**DOI:** 10.1186/1471-2334-13-296

**Published:** 2013-07-01

**Authors:** Kensuke Takahashi, Motoi Suzuki, Le Nhat Minh, Nguyen Hien Anh, Luu Thi Minh Huong, Tran Vo Vinh Son, Phan The Long, Nguyen Thi Thuy Ai, Le Huu Tho, Konosuke Morimoto, Paul E Kilgore, Dang Duc Anh, Koya Ariyoshi, Lay Myint Yoshida

**Affiliations:** 1 Department of Clinical Medicine, Institute of Tropical Medicine, Nagasaki University, 1-12-4 Sakamoto, Nagasaki, Japan; 2 National Institute of Hygiene and Epidemiology, 1 Yersin, Hai Ba Trung, Hanoi, Vietnam; 3 Khanh Hoa General Hospital, 19 Yersin, Loc Tho, Nha Trang, Khanh Hoa, Vietnam; 4 Khanh Hoa Health Service, 3 Han Thuyen, Nha Trang, Khanh Hoa, Vietnam; 5 Wayne State University, 5057, Woodward Avenue #3101, Detroit, MI, USA

**Keywords:** Community-acquired pneumonia, Aetiology, Incidence, Ageing, Southeast Asia, Vietnam, Viral pneumonia, Nasopharyngeal swab, Multiplex PCR

## Abstract

**Background:**

Lower respiratory tract infection (LRTI) including Community-acquired pneumonia (CAP) is a common infectious disease that is associated with significant morbidity and mortality. The patterns of aetiological pathogens differ by region and country. Special attention must be paid to CAP in Southeast Asia (SEA), a region facing rapid demographic transition. Estimates burden and aetiological patterns of CAP are essential for the clinical and public health management. The purposes of the study are to determine the incidence, aetiological pathogens, clinical pictures and risk factors of community-acquired pneumonia (CAP) in the Vietnamese adult population.

**Methods:**

A prospective surveillance for hospitalised adult CAP was conducted in Khanh Hoa Province, Central Vietnam. All adults aged ≥15 years with lower respiratory tract infections (LRTI) admitted to a provincial hospital from September 2009 to August 2010 were enrolled in the study. Patients were classified into CAP and non-pneumonic LRTI (NPLRTI) according to the radiological findings. Bacterial pathogens were identified from sputum samples by the conventional culture and polymerase chain reaction (PCR) for *Streptococcus pneumoniae*, *Haemophilus influenzae*, and *Moraxella catarrhalis*; 13 respiratory viruses were identified from nasopharyngeal specimens by PCR.

**Results:**

Of all 367 LRTI episodes examined, 174 (47%) were CAP. Older age, the presence of underlying respiratory conditions, and higher index score of smoking were associated with CAP. The one-year estimated incidence of hospitalised adult CAP in our study population was 0.81 per 1,000 person years. The incidence increased considerably with age and was highest among the elderly. The case fatality proportion of hospitalised CAP patients was 9.8%. Among 286 sputum samples tested for bacterial PCR, 79 (28%) were positive for *H*. *influenzae*, and 65 (23%) were positive for *S*. *pneumoniae*. Among 357 samples tested for viral PCR, 73 (21%) were positive for respiratory viruses; influenza A (n = 32, 9%) was the most common.

**Conclusions:**

The current adult CAP incidence in Vietnam was relatively low; this result was mainly attributed to the young age of our study population.

## Background

Community-acquired pneumonia (CAP) is a major cause of morbidity and mortality in adults [1,2]. Worldwide, lower respiratory tract infections (LRTIs) including pneumonia are the third highest cause of death in adults, and 1.8 million people aged ≥15 years die from pneumonia every year [3]. Studies have shown that the risk of CAP increases with age and is highest among the elderly, indicating that the burden of this health risk grows as the global population ages [4-6]. Special attention must be paid to CAP in Southeast Asia (SEA), a region facing rapid demographic transition [7].

From the clinical and public health perspectives, estimates of the overall health care burden and aetiological patterns of CAP are essential [1,2]. According to a recent systematic review, the patterns of aetiological pathogens differ by region and country [8]. In SEA, the overuse of antibiotics may be associated with different pathogen distributions [9-12]. As a result of these differences, the empirical antimicrobial therapy and vaccination (e.g., 23-valent pneumococcal polysaccharide vaccine and influenza vaccine) programmes recommended in Western countries may not be directly applicable to the adult population in this region. Complicating the issue, available data on the epidemiology of CAP are seriously limited in SEA countries, including Vietnam [11,12].

The major limitation in conducting a CAP study in resource-limited settings is the lack of sensitive diagnostic tools and standardised clinical data [12,13]. The recent development of improved microbiological methods, including polymerase chain reaction (PCR), may overcome this limitation. One of the advantages of PCR for respiratory pathogens is its ability to identify pathogens in patients who have already received antimicrobial therapy [14,15]. Only a few studies have applied this method to the study of CAP in SEA [12], and no study on adult CAP has been reported from Vietnam.

The aims of this prospective surveillance were 1) to estimate the incidence of hospitalised adult CAP, 2) to determine the causative viral and bacterial pathogens of CAP, and 3) to describe the clinical features of CAP in Vietnam and 4) to identify the risk factors that influence the severity of LRTI among Vietnamese adults. We used standardised case definitions and applied comprehensive and sensitive molecular diagnostic techniques.

## Methods

### Study population and hospital

Nha Trang is the capital city of the Khanh Hoa Province in South-Central Vietnam. Temperatures are high throughout the year (average 26°C), and the rainy season is from September through December. According to the Vietnam Population and Housing Census and our previously conducted population-based survey, the total mid-year pop-ulation in the city in 2009 was 392,279, with76% of ≥15 years and 6.4% of ≥ 65 years old [16,17]. Influenza vaccine and pneumococcal polysaccharide vaccine had not been included in the national immunisation programme at the time of the study. The introduction of the *H*. *influenzae* type b (Hib) conjugate vaccine for all infants aged 2, 3, and 4 months did not take place until May 2010.

The Khanh Hoa General Hospital (KHGH) is a pro-vincial-level referral hospital that provides primary and tertiary care for residents. According to our previous survey, the hospital served as the sole provider of hospital care for severe diseases in Nha Trang City [16].

### Patient enrolment

All physicians and infectious disease specialists screened adult patients hospitalised with acute LRTI. Our target population was all patients aged ≥15 years residing in Khanh Hoa Province who were hospitalised with any signs of LRTI from September 1, 2009, through August 31, 2010. A patient was classified as having LRTI if presenting to the hospital with at least two of the following symptoms: (1) fever and/or cough (2) fast and/or difficulty of breathing, or (3) any additional severe symptoms including respiratory rate over 30 per minute, SpO2 under 90%, systolic blood pressure under 90 mmHg, pulse rate more than 130/min, white blood cell count over 20,000 or under 4,000 cells/μL, CRP over 20 mg/dl, dehydrated, altered consciousness and other worse general status. Patients were excluded from the study if they had been hospitalised within the previous week, or have been diagnosed other than LRTI, or if no interpretable chest X-ray was obtained. These criteria were based on criteria in previous studies of CAP and modified considering local settings [18].

Demographic and clinical information were collected using a standardised form. The forms were filled out by the hospital physicians, and the data were verified by one dedicated research clinician and one research assistant. Malnutrition was defined as clinically apparent weight loss or skinny and pale appearance accompanied with disability or reduction of eating for more than 7 days. Status of human immunodeficiency virus (HIV) infection was not routinely tested but positive results in previous test were noted. Blood, sputum, and flocked nasopharyngeal swab (NPS) specimens were obtained from the participants immediately after admission and completion of an informed consent form. Chest X-rays were taken within 48 hours of admission.

### Laboratory testing

The clinical specimens were immediately transported to the hospital laboratory. Gram staining was performed on all sputum, and the specimen quality was evaluated according to Geckler’s category [19]. Conventional bacterial culture was performed by trained laboratory technicians. To each of the NPS specimens, 800 μl of normal saline (NS) was added for processing; then, 140 μl of this fluid was transferred to a medium containing skim milk, tryptone, glucose, and glycerine (STGG media). Another 200 μl of the NPS-saline solution was added to viral transfer media (VTM). Residual sputum samples and VTM were stored at −80°C in the hospital laboratory and transported on ice pack to the Institute of Tropical Medicine at Nagasaki University, Japan every three months for further molecular tests. The sputum samples were tested by multiplex PCR, which is a conventional PCR with mixing three sets of pathogen specific primers to identify three typical pathogenic bacteria: *Streptococcus pneumoniae*, *Haemophilus influenzae*, and *Moraxella catarrhalis*. Viral nucleic acid was extracted from VTM using the QIAamp viral RNA extraction kit and tested for 13 previously described respiratory viruses: influenza A/B (FLUA, FLUB), respiratory syncytial virus (RSV), Human adenovirus (HAdV), Human rhinovirus (HRV), Human bocavirus (HBoV), Human metapneumovirus (HMPV), Human parainfluenza virus (HPIV) type 1–4, and Human coronavirus OC43/229E (HCoV) [16].

### Definitions of pneumonia

Patients were classified into either CAP or non-pneumonic lower respiratory tract infections (NPLRTI) according to the findings of the chest X-rays. As there are no common criteria to interpret adult chest X-ray, the World Health Organization’s guideline for childhood pneumonia were modified to standardize the interpretation algorithm [20]. A senior pulmonologist and two physicians who were blinded to the clinical course interpreted all chest X-ray films independently. A case was categorised as CAP if two or more evaluators agreed on the presence of consolidation. The severity of cases was assessed using CURB65 and Pneumonia Severity Index (PSI) scores [21].

### Estimation of the incidence of CAP

To ensure all CAP cases are counted in the incidence estimation, we obtained the hospital admission database with International Classification of Diseases, 10th revision (ICD-10) coding along with age, gender, date of admission and discharge and address codes. The cases of hospitalised ICD-10 coded pneumonia were defined by ICD-10 codes J10–J18 (ICD-10 coded pneumonia).

Let *L*_*a *_denote the number of actively enrolled cases of LRTI and C_a_ denote the number of actively enrolled CAP cases with chest X-ray confirmation. Let *C*^*d *^denote the number of cases with ICD-10 coded pneumonia in the hospital database. Amongst *C*^*d *^there were some cases which had been missed by the active enrolment and hence no chest X-ray was available to determine whether they attributed to the total incidence of CAP. Among those cases which were included in both *L*_*a *_and *C*^*d*^ (*L*_*a*_^*d*^) we determined the proportion (*r*), with respect to age group (*i*) and gender (*j*), which holds those cases with positive chest X-rays (*C*_*a*_^*d*^).

$$r\;\left[i,j\right]=C_{a}{}^{d}\left[i,j\right]/L_{a}{}^{d}\left[i,j\right]$$

We assumed that this proportion was the same for the rest of ICD-10 coded pneumonia cases and calculated the total number of cases with CAP (*C*_*T*_):

$$C_{T}\left[I,J\right]=C_{a}\left[I,J\right]+R\;\left[I,J\right]\times \left(C^{d}\left[I,J\right]-L_{a}{}^{d}\left[I,J\right]\right)$$

Age and gender specific incidence *I*[*i*, *j*] per 1,000 residents were calculated using population *P*[*i*, *j*];

$$I\;\left[i,j\right]=C_{T}\left[i,j\right]/P\left[i,j\right]\times 1,000$$

We calculated incidences in age group *i* of 15–29, 30–44, 45–64 and 75 years and over for gender *j* of male and female. To compare with other studies, additional estimates for other age groups were also done.

For calculation of the incidence, the population and patients were limited to Nha Trang residents because those who reside out of Nha Trang likely to go their own district level hospitals for mild symptoms.

### Definition of 2009pH1N1 influenza season

During the study period, 2009pH1N1 influenza outbreak occurred from Sep until Dec 2009. No cases with 2009pH1N1 influenza infection were detected from Jan 2010 until the end of the study according to on-going viral respiratory surveillance in this area [16,22]. Incidence of CAP in 2009pH1N1 influenza season was defined as cases detected from Sep to Dec 2009.

### Data analysis

The characteristics of patients with CAP and NPLRTI were compared using the chi-squared test for categorical variables, a two-tailed unpaired *t*-test for continuous variables and a nonparametric trend test for ordinal variables. Cases were excluded from each comparison if the relevant data were not recorded; imputations for missing values were not performed in our analyses. The 95% confidence intervals (CIs) for the incidence estimates were calculated using Wilson’s score method. The logistic regression analysis was applied to adjust for possible confounding factors. All statistical analyses were performed using Stata 12.0 (Stata Corp., USA).

### Ethics

This study was approved by the Institutional Review Board (IRB) of the National Institute of Hygiene and Epidemiology, Hanoi, Vietnam, and the IRB of the Institute of Tropical Medicine, Nagasaki University, Nagasaki, Japan. Informed consent was obtained in accordance with international guidelines.

## Results

### Demographic data and clinical presentation

**Figure 1:**
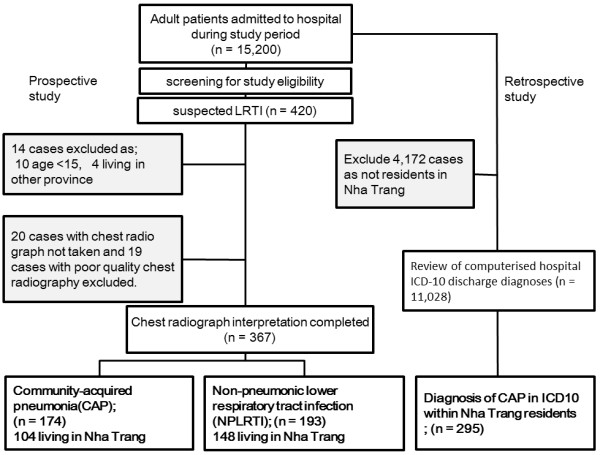
**Flow chart of case enrolment and allocation.** ICD-10; International Classification of Diseases, 10th revision. Left branch indicates active surveillance based on admission diagnosis of LRTI and 46 cases were excluded. Numbers of patients living in Nha Trang were used to calculate incidence of pneumonia. Right branch shows retrospective surveillance based on discharge diagnosis.

A total of 367 LRTI episodes were found in 359 patients who were actively enrolled in the study. Five patients had two episodes each, and one patient had four episodes. A total of 174/367 (47%) were CAP, and the rest were NPLRTI (Figure 1). Median age (range) of enrolled cases, CAP and NPLRTI were 42 (15–97), 50 (15–97) and 34 (16–95) respectively. Table 1 shows a comparison of demographic and clinical characteristics by endpoints. Older age ≥ 65 years, history of tuberculosis, malnutrition and higher smoking index (e.g. number of cigarettes per day multiplied by smoking year) were associated with CAP.

**Table 1:** Table 1 The demographic and clinical characteristics of study patients

**Characteristics**	**CAP**	**NPLRTI**	P value*
	**(n** = **174)**	**(n** = **193)**	
Male sex (%)	91 (52)	93 (48)	0.4
Age ≥ 65 years (%)	57 (33)	39 (20)	0.006
Presence of underlying conditions^†^ (%)	115 (66)	99 (51)	0.004
HIV infection	4 (2)	3 (2)	0.6
History of tuberculosis (%)	13 (7)	3 (2)	0.006
Asthma (%)	11 (6)	13 (7)	0.9
Malnutrition (%)	30 (17)	11 (6)	<0.001
Cerebrovascular disease (%)	17 (10)	15 (8)	0.5
Living with children aged <5 years (%)	55 (32)	60 (31)	1.0
Cigarette smoking history			
Current/past smoker (%)	78 (45)	70 (36)	0.1
Current/past smoker, male (%)	74 (81)	65 (70)	0.07
Smoking index^‡^, mean ± SD	120 ± 219	63 ± 137	0.003
0–49 (%)	107 (61)	143 (74)	0.014
50–99 (%)	14 (8)	10 (5)	
≥100 (%)	53 (30)	40 (21)	
Symptomatic period^§^, mean days ± SD	2 ± 5	2 ± 4	0.5
Preceding antibiotics use/data available (%)	35/147 (24)	45/158 (28)	0.4
Pneumonia severity index^∫^			
I–II (%)	37/62 (60)	36/48 (75)	0.09
III–V (%)	25/62 (14)	12/48 (6)	
CURB65 score (n = 124) ^∫^			
0 (%)	10/70 (14)	20/54 (37)	0.0043
1 (%)	39/70 (56)	25/54 (46)	
2–4 (%)	21/70 (30)	9/54 (17)	
Length of hospital stay, median days ± SD	8 ± 7	5 ± 7	0.016
Outcome at discharge			
Survived	145 (83)	185(96)	<0.001
Transferred to other hospital	12 (7)	3 (2)	
Deceased	17 (10)	5 (3)	

* Chi-squared tests were used for binary categorical variables, non-parametric trend test were used for ordinary categorical variables and two-tailed unpaired t-tests were used for numerical variables.

† Underlying conditions include malnutrition, chronic diarrhoea, heart disease, chronic lung disease, neuro-nervous disease, renal disease, thalassemia, liver disease, chronic heart failure, cerebrovascular disease, active tuberculosis, and COPD.

‡ The number of cigarettes smoked per day X total duration in years.

§ A period from onset to admission.

∫ Pneumonia Severity Index (PSI): Scores range from approximately 10 to 250; higher scores indicate more severe disease. Patients with PSI scores of 70 or fewer points are classified as classes I to II. CURB65 is the scoring system to add one point for each of confusion, urea >7 mmol/l, respiratory rate >30/min, low systolic (90 mmHg) or diastolic (60 mmHg) blood pressure, and age >65 years

The majority of both CAP and NPLRTI patients were classified as mild cases; 66% (73/110) were PSI classes I or II, 76% (94/124) were CURB65 0 or 1. The PSI and CURB65 scores were higher in patients with CAP than in patients with NPLRTI. The median length of hospitalisation was significantly longer in patients with CAP. Among 174 CAP cases, 17 patients died (case fatality proportion = 9.8%); eight were aged < 65 years (CFP = 7.3%), and nine were aged ≥ 65 years (CFP = 15.8%). Six CAP patients and two NPLRTI patients were transferred to a local tuberculosis hospital. Malnutrition was observed more in deceased patients than in those who survived (75.6% vs. 24.4%, p < 0.001). A total of 26% (80/305) of the patients had received antibiotic treatment within seven days before hospitalisation. Younger adults, aged 15–64 years, took antibiotics before admission more frequently than elderly patients ≥ 65 years (76/212, 36% vs. 4/93, 4%, p < 0.001), however, taking antibiotics did not affect the risk of developing pneumonia in both age groups in the multivariate logistic regression analysis (P = 0.9).

### Incidence of CAP

The number of ICD-10-coded pneumonia cases as identified from the hospital admission database was 295, which was three times higher than that of actively enrolled CAP among Nha Trang residents. This group was significantly older than the actively enrolled CAP group. Amongst those which were identified by ICD-10 coded pneumonia, there was no significant difference in the mean length of hospital stay by each age group between actively enrolled cases and not enrolled cases.

**Figure 2:**
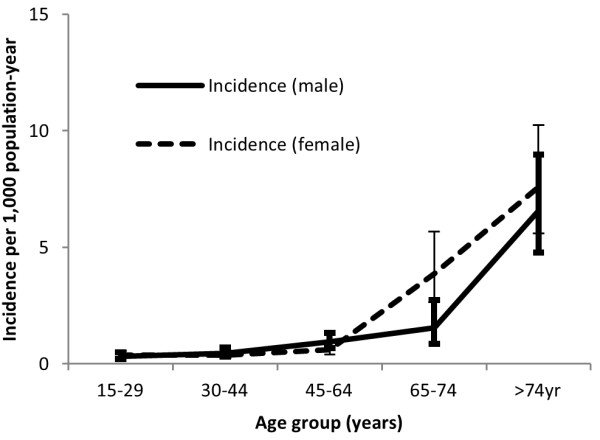
**The incidence of hospitalised pneumonia per 1**,**000 persons by age group and gender.** Incidence among male population were showed in solid line and those among female population were showed in dashed line. 95% confidence interval of each points were showed in vertical lines.

The estimated age group-specific incidences of CAP are shown in Table 2. The incidence of CAP in the elderly aged ≥ 65 years was 4.6 per 1,000 person years (PY) (95% CI: 3.8–5.5), which was ten times higher than the incidence in the younger age group; 0.48 (0.41 – 0.57). Figure 2 shows sex-specific incidence rates for each age group. The incidence of CAP in females was higher than that of males for ages 45–64 years, whereas no gender difference was observed in the one-year estimated incidence for total adult population.

**Table 2:** **Incidence of CAP among adults by gender and age group**, **Nha Trang**, **Vietnam**

**Characteristics**	Total estimated CAP^*^		**Actively enrolled CAP**^**†**^		**Population**
	**N**	**Incidence rate**^**‡**^	**N**	**Incidence rate**	
		**(95% CI**^**§**^**)**		**(95% CI)**	
Total	241	0.81	106	0.38	299,000
		(0.71–0.91)		(0.29–0.43)	
Sex					
Male	120	0.82	55	0.38	140,321
		(0.68–0.98)		(0.29–0.49)	
Female	119	0.78	51	0.34	158,679
		(0.65–0.94)		(0.26–0.44)	
Age					
group (year)					
15–29	37	0.34	23	0.21	110,288
		(0.24–0.46)		(0.14–0.31)	
30–44	40	0.42	26	0.27	96,427
		(0.31–0.57)		(0.18–0.40)	
45–64	54	0.80	21	0.31	67,178
		(0.62–1.05)		(0.20–0.48)	
65–74	37	2.67	15	1.08	13,885
		(1.93–3.67)		(0.66–1.78)	
≥75	78	6.95	21	1.87	11,222
		(5.57–8.67)		(1.22–2.86)	

* Number of cases estimated using ICD-10 coded hospital database.

† Radiological confirmed pneumonia enrolled in our active surveillance.

‡ Per 1,000 person years.

§ Confidence interval.

### Aetiology

A total of 323 sputum samples were obtained from actively enrolled 323 cases. Of the 289 tested by bacterial culture, 43 (15%) were positive. Of the 286 tested by PCR, 132 (46%) were positive for at least one bacterial pathogen, including 19 samples with dual infection and four with triple infection. In culture, *S*. *aureus* and *M*. *catarrhalis* were the most frequently isolated, followed by *S*. *pneumoniae*, whereas in PCR, *H*. *influenzae* and *S*. *pneumoniae* were the most frequently detected, followed by *M*. *catarrhalis* (Table 3). 252 samples were tested for both bacterial culture and PCR (Additional file 1: Table S1). The concordance rate of these tests was 206/252 (81.7%), 190/252 (75.4%) and 238/252 (94.4%) for *S*. *pneumoniae*, *H*. *influenzae*, and *M*. *catarrharis*, respectively. The numbers of the positive PCRs with negative bacterial cultures were more than those of positive cultures with negative PCRs for all three bacteria. Overall, among 323 samples tested for either culture and/or PCR, 149 (46%) were positive for some bacterial pathogens. The patterns of isolated bacteria were similar between CAP and NPLRTI. These patterns were also similar between age groups, except that *P*. *aeruginosa* was isolated more frequently in the elderly (p = 0.031). Blood cultures were performed for all enrolled cases; *Klebsiella* was isolated in one case, and *S*. *aureus* was isolated in two cases. According to Gram staining, only 12% (n = 24/208) of evaluated sputum samples showed acceptable quality (4 or 5 in Geckler’s category). Even among these 24 quality-assured sputum samples, only 13% (n/N = 3/24) turned out to be positive by culture, whereas approximately 52% (n/N = 11/21) were positive by PCR: *S*. *pneumoniae* (n = 5), *H*. *influenzae* (n = 4), and *M*. *catarrhalis* (n = 2).

**Table 3:** The bacterial and viral aetiology of the enrolled patients

	**Number of patients (%)**				
		**Case category**		**Age group**	
	**Total**	**CAP**	**NPLRTI**	**15–64 years**	**≥ 65 years**
	**(n = 323)**	**(n = 154)**	**(n = 169)**	**(n = 237)**	**(n = 93)**
Bacterial culture No. tested	289	135	154	209	80
No. positive	43(15)	22(16)	21(14)	31(15)	12(15)
*S*. *pneumoniae*	8(3)	4(3)	4(3)	6(3)	2(3)
*M*. *catarrhalis*	5(2)	3(2)	2(1)	4(2)	1(1)
*H*. *influenza*	10(3)	5(4)	5(3)	8(4)	2(3)
*S*. *aureus*	11(4)	3(2)	8(5)	9(4)	2(3)
*P*. *aeruginosa*	4(1)	4(3)	0	1(0)	3(4)
*K*. *pneumonia*	5(2)	3(2)	2(1)	3(1)	2(3)
Bacterial PCR No. tested	286	135	154	209	80
No. positive	132 (46)	61 (45)	71 (47)	104 (46)	28 (45)
*H*. *influenza*	79 (28)	37 (27)	42 (28)	63 (28)	16 (26)
*S*. *pneumonia*	65 (23)	31 (23)	34 (23)	51 (23)	14 (23)
*M*. *catarrhalis*	15 (5)	8 (6)	7 (5)	11 (5)	4 (6)
Respiratory viruses No. tested	357	167	190	264	93
No. positive	73 (20)	27 (16)	46 (24)	59 (22)	14 (15)
Influenza A	32 (9)	10 (6)	22 (12)	26 (10)	6 (6)
Influenza B	13 (4)	5 (3)	8 (4)	13 (5)	0
Rhinovirus	22 (6)	9 (5)	13 (7)	16 (6)	6 (6)
Adenovirus	3 (1)	3 (2)	0	3 (1)	0
RSV	4 (1)	1 (1)	3 (2)	2 (1)	2 (2)
Viral-bacterial co-infection	57 (18)	26 (17)	31 (19)	45 (19)	12 (14)

Of 357 NPS samples tested for respiratory viruses, 73 (20%) were positive for some viruses, including one with dual infection of FLUA and HAdV. FLUA was the most frequently detected, followed by HRV (Table 3). The patterns of detected viruses were similar between CAP and NPLRTI and between age groups, except that Influenza B was observed more frequently in younger adults (p = 0.029).

**Figure 3:**
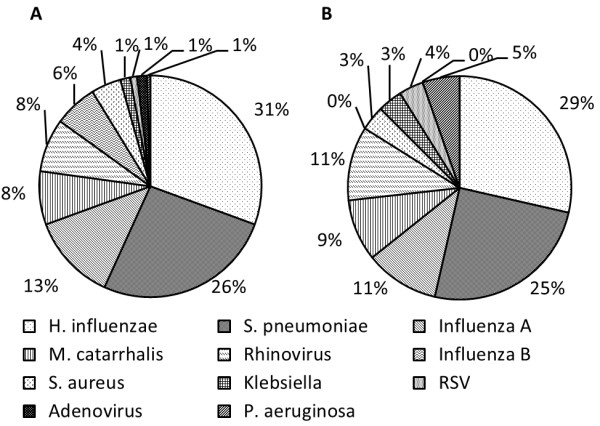
**The proportion of viral and bacterial agents identified from the enrolled patients by age group.** Pathogens detected in sputum and/or nasopharyngeal swabs of patients with LRTI. Patients of 15 to 64 years old were presented in left **(A)** and those over 65 years old were presented right **(B)**.

In 245 cases with samples completed for both bacterial and viral tests, pathogens were identified in 151 (62%). In 26 (11%) of the cases, both virus and bacterial pathogens were detected, but there was no significant association between the detection of virus and of bacteria. HMPV, HBoV, PIV1-4, and HCoV were not detected in any of our cases. The distribution of identified pathogens in each age group is shown in Figure 3. The mean duration from onset to admission was significantly longer in cases with bacterial infection (mean ± SD: 4.2 ± 5.8 days, n = 123, p = 0.013) compared with that of cases without bacterial infection (2.7 ± 3.1 days, n = 122)

### Impact of the 2009 influenza pandemic

**Figure 4:**
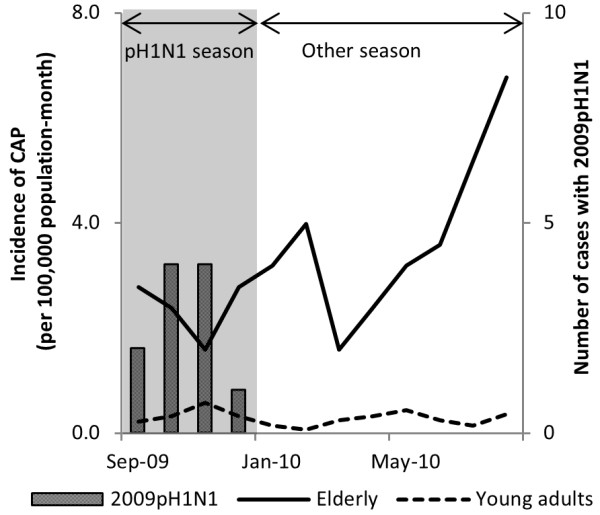
**The monthly incidence of CAP amongst elderly** ≥ **65 years and young adult 15**–**64 years and number of 2009pH1N1 influenza infections.** The 2009pH1N1 season was defined between September 2009 and December 2009. The bars indicate number of 2009pH1N1 detected in our study. The estimated monthly incidences per 100,000 population amongst elderlies ≥ 65 years old were showed in solid line, those amongst young adults in dashed line.

In Vietnam, the first case of pandemic influenza A (H1N1) 2009 was reported on May 31, 2009 [23]. In Nha Trang, the first case was reported in early July 2009, and the number of reported cases reached a peak in October and declined until December. After December 2009 until August 2010, the end of current study, 2009pH1N1 was not detected. During the pandemic, the monthly incidence of hospitalised CAP amongst younger adults significantly increased 1.6 times more than in the other seasons (0.53 per 10,000 population-month, 95%CI 0.40-0.68 vs. 0.32, 0.25-0.41), whereas the incidence among the elderly decreased (2.8, 1.9 – 4.0 vs. 4.3, 3.4 – 5.3). Subsequently, the total incidence of CAP remained stable (0.71, 0.57 – 0.88 vs. 0.63, 0.53 – 0.74) despite the influenza pandemic (Figure 4).

## Discussion

### Incidence of CAP

Our study revealed that the one-year estimated incidence of hospitalised CAP among adults aged ≥15 years in Central Vietnam was 0.8 per 1,000 PY. This figure was, in fact, lower than that of similar studies with chest X-ray-confirmed pneumonia in the Western world, such as studies in the USA (patients aged > 45 years: 16 per 1,000 PY vs. 1.8 per 1,000 PY in our study) [24], in Spain (patients aged ≥15 years: 1.2-1.6 per 1,000 PY vs. 0.8 per 1,000 PY) [4,6], in Germany (patients aged ≥18 years: 3.7 per 1,000 PY) [25], and in Finland (patients aged ≥ 50 years: 6.3 per 1,000 PY vs. 2.1 in our study aged ≥ 50 years) [26].

Several factors explain this difference. First, the definition of pneumonia varies by study [25]. Certain studies used chest X-ray findings to determine pneumonia [4,6,26], whereas others used clinically defined criteria or simply relied on reported cases at the sentinel sites [24,25]. In our study, we carefully defined pneumonia using stringent criteria based on chest X-ray findings; with these criteria, only 45% of clinically diagnosed LRTIs exhibited consolidation on chest X-rays. Thus, our figures are conservative estimates. Second, our study targeted hospitalised CAP and did not include cases treated at the outpatient department or at neighbouring clinics. Since our hospital is only tertiary hospital in this area, there was a concern that only severe cases were admitted to this hospital. However, the proportions of mild cases with PSI classes I or II and CURB65 score 0 or 1 were similar to those reported by Capelastegui (CURB65 0–1 69.8%, incidence of CRP 3.1 per 1,000PY) [27] and Gutiérrez (PSI classes I and II 54.2%, incidence of CAP 1.2 per 1,000PY) [6]. We therefore believe that a selective bias towards severe patients is not a major factor explaining the low incidence rate although we need to collect the information about health care seeking behaviour within the community to determine the actual incidence of CAP. Third, the age distribution is substantially different; the proportion of the elderly in Vietnam is lower than those in Western counties. Although the one-year estimated incidence of CAP in Spain was higher than our estimates, their age group-specific incidences were compatible with our estimates: 0.7, 1.1, 2.4, and 5.3 per 1,000 PY for the age groups of 15–44, 45–64, 65–74, and over 75 years, respectively [6]. This finding indicates that the relatively low one-year incidence of CAP was mainly attributable to differences in the population structures. A similar large-scale study conducted in Thailand also showed a slightly higher incidence, but that study included children under 5 years old with a very high incidence rate of pneumonia [12].

### Aetiology of CAP

*H*. *influenza*e and *S*. *pneumoniae* are known to be common causes of adult CAP. Similarly, these pathogens were most frequently detected in adult patients with CAP in our study. Whereas *S*. *pneumoniae* was detected in 25% of samples, which is comparable with the results of other studies showing positivity of 9 to 48% [11], the proportion of positivity for *H*. *influenza*e (28%) in our study was substantially higher than previously reported: 2.7–6% in Sweden, Spain, and Thailand [14,28,29]. This difference may be because we used molecular techniques. There were some discrepancies between bacterial culture and PCR; more positive results were observed in PCR than culture but this tendency was compatible with previously published reports [15,30]. PCR methods are known to detect pathogens even just a few days after being exposed to antibiotics, which is difficult to detect with conventional bacterial culture [15]. We believe that molecular techniques are more appropriate for pathogen surveys because the results are less affected by pre-exposure to antibiotics. It is generally difficult to distinguish bacteria colonised in the oral cavity from bacteria as a causative pathogen. Quality assessment of sputum samples is therefore important; Gram staining can provide supplementary information. In this study, we simultaneously performed Gram staining and assessed the quality of sputum samples. Unfortunately, obtaining good quality sputum was found to be difficult. Only 11% turned out to be 4 or 5 in Geckler’s category. The proportion of positive samples and the pattern of pathogens did not significantly differ between good quality samples and those with lower quality. Furthermore, preceding antibiotic use might have affected the sensitivity of bacterial culture results. Although the clinical specimens were collected before the administration of antibiotics in the hospital, one-fourth of the patients in our study took antibiotics purchased at a local pharmacy prior to their admission. When all of these factors are considered, careful interpretation of the aetiology results is necessary.

Respiratory viruses were detected in 21% of adult CAP cases. This value is comparable with the results of other studies from European countries, which showed that 12–32% of hospitalised CAP patients were viral positive [31-33]. Influenza A and HRV are the leading pathogens of childhood acute respiratory infections, but positivity for these viruses was much lower in adults [12,32-34]. Recent evidence indicates that HRV can cause pneumonia in very young children and in severely immunocompromised patients, but the role of HRV in pneumonia among immunocompetent adults remains controversial [32,34]. Although our finding could not confirm lower respiratory infection with HRV, the high prevalence of HRV detected from nasopharynx samples in adult CAP cases suggests the involvement of HRV as a cause of pneumonia [34]. RSV is also not uncommon in the adult population, as Falsey reported that RSV infection accounted for 11% of pneumonia among elderly and at-risk adults in the winter season in the USA [35]. In our study, RSV positives accounted for 6% of hospitalised CAP patients during the RSV season, July to November in Central Vietnam [16].

Evidence in several studies indicates that viral infection is an important trigger of secondary bacterial infection; previous viral infection might impair mucosal barriers in the respiratory system and make the host susceptible to bacterial infection [36,37]. Furthermore, we have previously reported that the bacterial burden of *S*. *pneumoniae* in nasopharyngeal swabs was significantly higher in virus co-infected patients [17]. In the current study, patients with single bacterial infection had a significantly longer duration prior to hospital admission compared with patients with virus infection. It is plausible that patients with single bacterial infection have had a previous viral infection, which had resolved by the time of hospitalisation.

### Influence of HIV/AIDS patients

We included 7 cases with known HIV infection. While some similar studies in other regions excluded immuno-suppressed patients [29] or restricted to previously well patients [18], others did not exclude these patients [4,6,12,24,26]. Our concern was that the regional variation in the prevalence of HIV infection could affect the incidence of pneumonia. However, even excluded these patients the incidence of CAP was not changed much (0.81/1,000 population-year, 95%CI 0.71-0.91). The aetiology of pneumonia amongst patients with HIV/AIDS may differ, but because of limited laboratory resource, we couldn’t test for additional pathogens like *P*.*jirovecci*, cytomegarovirus or criptococcus. In one HIV positive case, co-infection of *S*. *pneumoniae* and adenovirus was detected by bacterial culture and viral PCR. One single *H*. *influenzae* infection, two single *S*. *pneumoniae* infections and one co-infection with *S*. *pneumoniae* and *H*. *influenzae* were detected from bacterial PCR, and no pathogen was detected from the other 3 cases. Thus the aetiology in HIV infected cases was not apparently different from those without HIV infection.

### Limitations of the study

Despite the prospective survey, to which one dedicated clinician and one research assistant were attached for checking those enrolments, a considerable proportion of ICD10-coded-pneumonia was not enrolled. This situation was mainly due to certain pneumonia patients who were un-diagnosed on admission, and this tendency was particularly observed in elderly patients, who often lacked symptoms of acute respiratory infection on admission. We therefore identified all hospitalised CAP using the admission records and went on to estimate the incidence, adjusting for enrolment. In the current study, we had to make an assumption that the proportion of X-ray confirmed CAP was same in the whole cases of ICD-10 code pneumonia. This assumption may not be true but we could not review chest X-rays amongst retrospectively identified ICD-10 coded pneumonia cases, because their chest X-rays were kept by patients, not stored in the hospital. The difficulty still remains in distinguishing nosocomial pneumonia from CAP in ICD-10 coded pneumonia cases. However, the length of hospital stay amongst patients with nosocomial pneumonia is expected to be longer than those with CAP [38]. The similarity of the length in our study groups indicates that the majority of ICD-10 coded pneumonia cases were not nosocomial pneumonia. Another limitation comes from difficulties in collecting good-quality sputum specimens, as was discussed above.

### Population ageing and CAP

**Figure 5:**
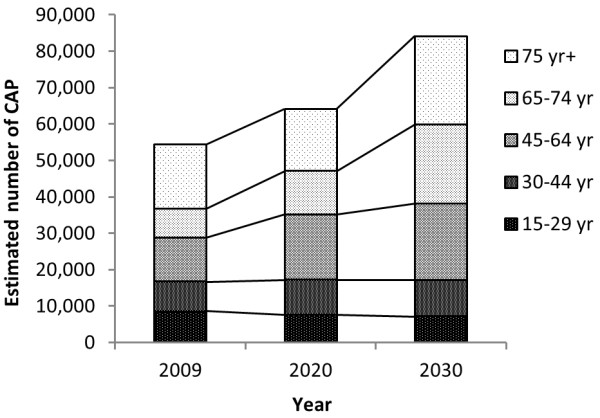
**The predicted number of adult CAP patients in Vietnam in 2009, 2020, and 2030.** The age-group specific number of CAP patients was estimated using the predicted population number [7] and our age group-specific incidence estimates.

Our study revealed that the one-year estimated incidence of CAP was relatively low in the current adult population in Vietnam, but we showed that both the incidence and the case fatality ratio drastically increased with age and were highest among the elderly. This observation indicates that the disease burden will rapidly increase as the population gets older. According to the United Nations Population Fund (UNFPA), the Vietnamese elderly population (persons aged ≥ 65 years) will reach 11 million (11%) in 2030, which is almost double the 2009 percentage [7]. According to our estimates, the annual number of hospitalised adult CAP patients in all of Vietnam will increase from 2009 to 2030 by 55% in people aged ≥15 years and by 79% in those aged ≥ 65 years (Figure 5). Early intervention, such as vaccination against *S*. *pneumoniae* and influenza for the adult population, is warranted to address this foreseeable problem in Vietnam and other countries in SEA.

## Conclusion

We revealed that age distributed incidence of CAP in Vietnam are similar to developed countries and estimated number of CAP patients would increase by rapid shift for aging population. *H*. *influenza*, *S*. *pneumoniae* and Influenza A virus were major pathogen detected.

## Competing interests

The authors declare that they have no competing interests.

## Authors’ contributions

KT participated in its design, collecting the data as a dedicated research clinician, analysed and interpreted the data and drafted the manuscript. MS helped interpreting and analysing data and revised the manuscript. LNM carried out molecular biological analysis. NHA carried out molecular biological analysis and coordinate the study. LTMH, TVVS and PTL participated in making enrolment criteria and collecting the data. NTTA carried out conventional biological studies. LHT coordinated the project and participated in its design. KM participated in its design and helped to interpret the data. PEK revised the manuscript. DDA participated in its design and coordination. KA participated in its design and helped to draft the manuscript. LMY participated in its design and coordination, helped to draft the manuscript. All authors read and approved the final manuscript.

## Pre-publication history

The pre-publication history for this paper can be accessed here:

http://www.biomedcentral.com/1471-2334/13/296/prepub

## Supplementary Material

Additional file 1: Table S1 The number of positive and negative cases between bacterial culture and PCR. Click here for file
