# Supplementary material for: The incidence and aetiology of hospitalised community-acquired pneumonia among Vietnamese adults: a prospective surveillance in Central Vietnam
Source: BMC Infect Dis. 2013 Jul 1;13:296. doi: 10.1186/1471-2334-13-296 (PMC3702433; doi:10.1186/1471-2334-13-296)
Supplement: Additional file 1: Table S1 — The number of positive and negative cases between bacterial culture and PCR. [file 1471-2334-13-296-S1.doc]

**Additional file 1: Table S1.** The number of positive and negative cases between bacterial culture and PCR.

| Bacterial culture | | Bacterial PCR | |
| --- | --- | --- | --- |
|  |  | Positive | Negative |
| *S.pneumoniae* | Positive | 5 | 3 |
|  | Negative | 43 | 201 |
| *H.influenzae* | Positive | 5 | 0 |
|  | Negative | 62 | 185 |
| *M.catarrharis* | Positive | 4 | 6 |
|  | Negative | 8 | 234 |
